# Renal Cell Carcinoma Metastasis to Meckel’s Cave Treated With Repeat Stereotactic Radiosurgery: A Case Report and Review of the Literature

**DOI:** 10.7759/cureus.16478

**Published:** 2021-07-19

**Authors:** John A Panizza, Mark B Pinkham, Matthew Foote, Mihir Shanker, Keith Horwood, Michael Huo

**Affiliations:** 1 Radiation Oncology, Princess Alexandra Hospital, Brisbane, AUS; 2 Radiation Oncology, University of Queensland, Brisbane, AUS; 3 Oncology, Greenslopes Private Hospital, Brisbane, AUS

**Keywords:** renal cell carcinoma, meckel’s cave, gamma knife, stereotactic radiosurgery, metastasis

## Abstract

Renal cell carcinoma (RCC) metastases to Meckel’s cave (MC) are a rare condition. To the best of our knowledge, we present the first case of an RCC metastasis to MC successfully treated on two consecutive occasions with stereotactic radiosurgery (SRS). A 57-year-old man presented with new-onset facial pain and numbness. Magnetic resonance imaging (MRI) revealed a lesion invading MC. He was treated with Gamma Knife SRS successfully, resulting in both symptomatic improvement and radiologic tumour regression. Thirteen months after treatment, he presented with a recurrence of trigeminal nerve symptoms. He was treated with hypofractionated SRS successfully, with a follow-up MRI revealing resolution of the disease. While RCC metastases to MC are a rare phenomenon, published literature to date recommends surgical resection in combination with radiotherapy and systemic therapy. Metastatic disease to MC has only been treated once before with radiosurgery alone. Our case demonstrates that repeat SRS is feasible and efficacious. This approach may be favourable in patients wishing to avoid risks of surgical resection, or for those with unresectable disease. Metastases of RCC to MC are a rare occurrence and typically present with facial pain and/or hypoesthesia. This case demonstrates that repeat radiosurgery may be an effective alternative to surgical resection.

## Introduction

Clear cell renal cell carcinoma (RCC) represents 85% of all primary renal malignancies and has a propensity to metastasise [1]. Typical sites of metastases are the lungs, bone, lymph nodes, liver, adrenal glands and brain. Approximately 8% of all patients with brain metastases come from RCC primaries [2]. RCC brain metastases portend a poor prognosis, associated with a two-fold increase in all-cause mortality [2]. Randomised data have demonstrated stereotactic radiosurgery (SRS) to be a preferred upfront treatment approach to whole-brain radiotherapy for patients with limited brain metastases, with high rates of local control and superior quality of life [3]. SRS involves the precise delivery of high doses of radiotherapy (typically in a single or up to five fractions) with a steep dose gradient to minimise dose to normal tissues [3].

Meckel’s cave (MC) is a dural pouch in the middle cranial fossa, which contains the trigeminal ganglion. Lesions invading the trigeminal ganglion within MC are usually benign tumours such as meningiomas or trigeminal schwannomas [1]. Metastases directly to MC are rare [1]; metastases invading from adjacent cavernous sinus are also possible but uncommon. To the best of our knowledge, there are three case reports describing four cases of RCC metastases to MC and each managed in different ways [1,2,4]. We hereby report a case of RCC metastasis to MC treated with repeat SRS, and review the literature for treatment recommendations and reported data applicable to this rare clinical situation.

## Case presentation

A 57-year-old man presented in April 2017 with non-specific headaches and a short history of left facial numbness and pain. He had no other visual nor cranial nerve symptoms such as ophthalmoplegia nor paraesthesia. He had a longstanding history of stage 4 clear cell RCC. He underwent a left nephrectomy in 2008 with metastatic recurrence demonstrated in 2010. He was initially treated with a right adrenalectomy and stereotactic radiotherapy to a lung lesion. In terms of systemic therapy, he received first-line sunitinib from June 2011 and dendritic cell vaccine therapy from July 2011, followed by nivolumab.

**Figure 1:**
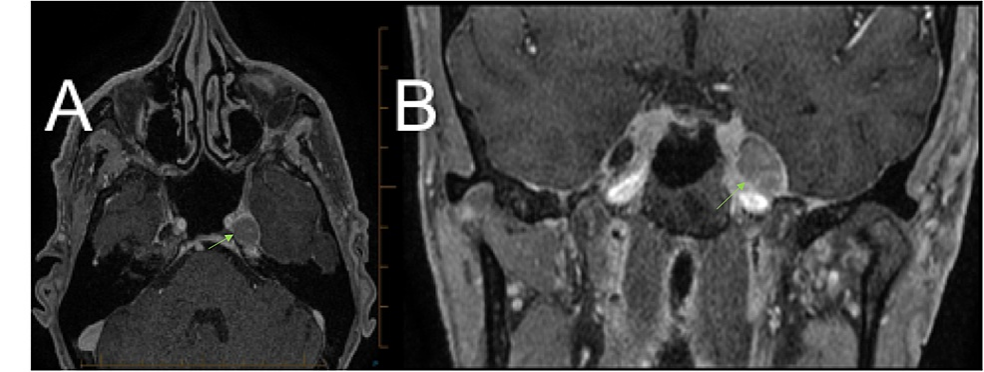
Magnetic resonance imaging brain (T1-weighted gadolinium-enhanced) taken on May 2017: (A) transverse and (B) coronal views demonstrating a left-sided lesion located in Meckel's cave with extension into the cavernous sinus.

Magnetic resonance imaging (MRI) of his brain revealed a left-sided lesion centered in MC with extension into the cavernous sinus. It measured 17 x 12 x 18 mm and enhanced with gadolinium. There was no intra-axial involvement. Based on the clinical features and imaging, the provisional diagnosis was metastatic disease. There was no prior MRI available for comparison, and prior staging compute tomography (CT) scans including cranial imaging did not reveal the lesion. A four-week-interval MRI demonstrated progressive growth in keeping with metastatic disease (Figure 1).

The patient was treated with Gamma Knife (GK) SRS to a dose of 20 Gy in one fraction prescribed to the 50% isodose in May 2017.

Clinical follow-up at two months post-GK SRS showed marked symptomatic improvement in facial pain and paraesthesia. The patient was able to cease analgesic medication. Radiological follow-up at six months post-GK SRS showed substantial tumour reduction on MRI; however, he had recently developed facial numbness and wasting of his muscles of mastication in keeping with a trigeminal nerve palsy. His lesion showed a partial response, reducing in size to 16 x 9 x 14 mm six months post-SRS with persistent residual enhancing changes seen one year following SRS.

**Figure 2:**
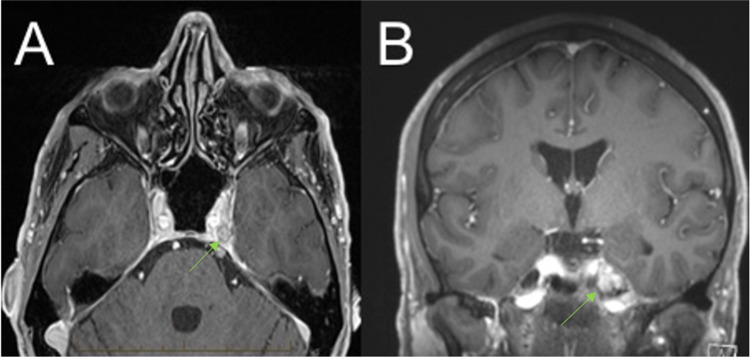
Magnetic resonance imaging brain (T1-weighted gadolinium-enhanced) taken on October 2018 – Follow-up imaging 13 months post-Gamma Knife stereotactic radiosurgery with (A) transverse and (B) coronal views demonstrating recurrent disease in the left Meckel's cave.

Just over 13 months post-GK SRS, he presented to clinic with a recurrence of facial pain, paraesthesia and new-onset diplopia consistent with a right sixth nerve palsy. MRI confirmed recurrent disease in the left MC with extension into the cavernous sinus (Figure 2). CT staging revealed three metastases progressing in the left lower lung, right hilum and left gluteal soft tissue mass. These signified progressive systemic disease.

 

**Figure 3:**
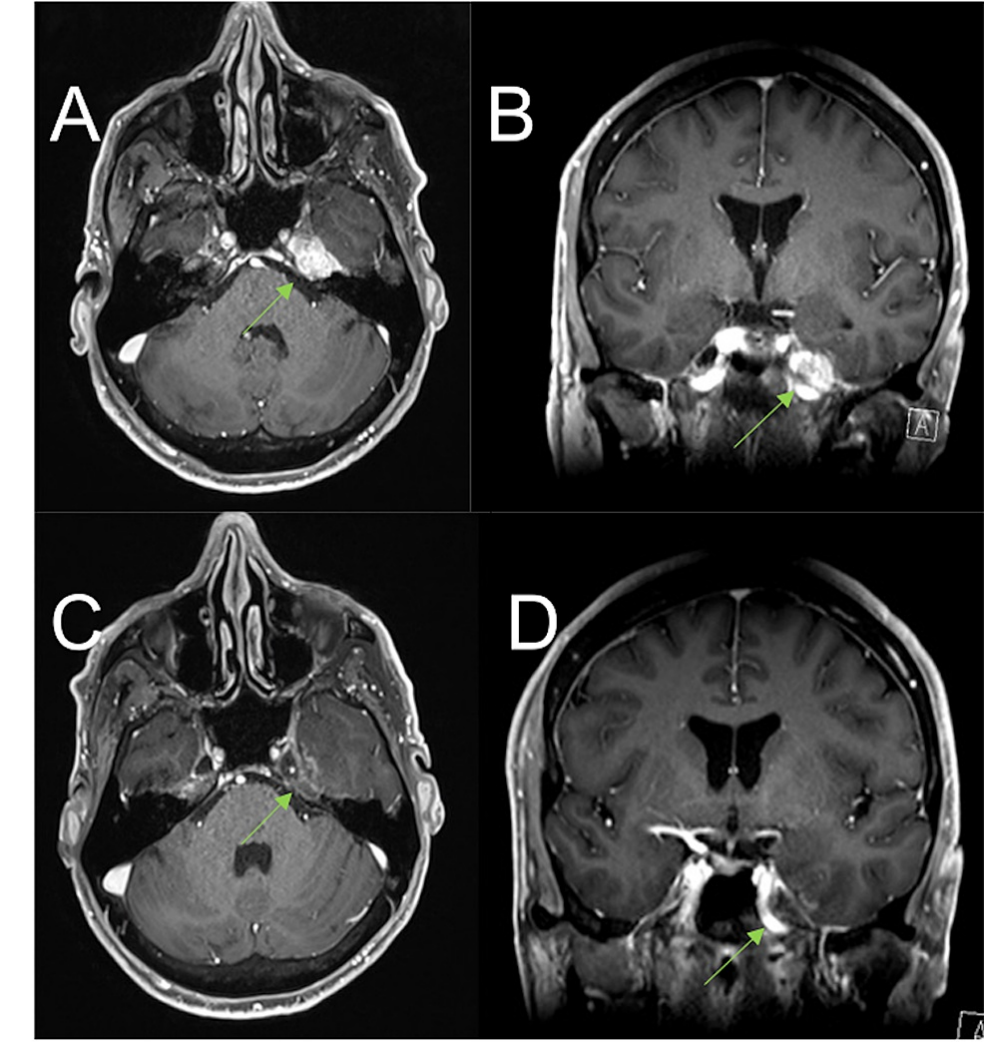
(A, B) MRI brain (T1-weighted gadolinium-enhanced) taken on October 2019. Follow-up study 29 months post-GK SRS and one month prior to repeat GK SRS with (A) transverse and (B) coronal views displaying progressive disease in left Meckel's cave extending to the cavernous sinus. (C, D) MRI brain (T1-weighted gadolinium-enhanced) taken on August 2020. Follow-up study nine months after repeat GK SRS with (C) transverse and (D) coronal views displaying mild early post-treatment effect enhancement in the adjacent temporal lobe. MRI, magnetic resonance imaging; GK, Gamma Knife; SRS, stereotactic radiosurgery.

The patient was recommenced on sunitinib, which showed an initial partial response, but subsequently, his systemic disease progressed again in October 2019 with enlargement of the MC lesion to 24 x 20 x 18 mm (Figure 3), a right lung mass and thoracic adenopathy. The result of a multidisciplinary team meeting was to retreat with SRS rather than surgery. He was treated with repeat GK SRS to the left cavernous sinus in November 2019 (30 months after initial SRS) to a dose of 30 Gy in five fractions at the 50% isodose line.

As of his MRI in August 2020 (nine months after repeat SRS), the MC metastasis was stable and post-treatment effect enhancement in the adjacent temporal lobe was noted (Figure 3). From his latest clinical follow-up in March 2021, his facial pain had completely resolved, while his paraesthesia had significantly improved but was still present. He continued to experience diplopia, managed with prism glasses. A repeat MRI in March 2021 confirmed stability of the treated lesion with some enhancing post-radiosurgical change and oedema.

## Discussion

To the best of our knowledge, no previous report has described a metastasis to MC that has been treated with radiosurgery on two occasions. RCC metastases to MC are a rare occurrence, with only four documented cases in the literature, each presenting with facial pain or hypoesthesia [1,2,4]. There is only one report of SRS without resection for metastatic disease from any primary site to MC, in a case of castration-resistant prostate cancer. GK was used to deliver a dose of 22 Gy, with radiosurgery providing a rapid improvement in symptoms with no adverse effects at one-month follow-up [5]. The patient declined further chemotherapy and died two months later, with no new neurologic or GK side effects noted [5].

Treatment options for metastatic disease to MC include surgical resection, radiotherapy or SRS and/or systemic therapy [1,4]. Surgical resection of tumours affecting MC are often not feasible and/or associated with significant morbidity, contributing to the decision in this case to avoid surgery. Most MC lesions can be accurately diagnosed by clinical context and imaging. However, if biopsy is required for definitive diagnosis, a focused transcranial approach via middle fossa craniotomy is preferred over minimally invasive methods as it provides greater diagnostic yield and lower risk of compressive trigeminal neuropathy secondary to RCC bleed [2].

RCC is considered radioresistant to traditional fractionated radiotherapy, whereas SRS is a highly effective option for local control [6]. In this case, the patient’s RCC was treated with GK on two separate occasions, both treatments providing symptomatic relief. Most recent follow-up at the time of writing was approximately 47 months following initial diagnosis of the MC metastasis, which is far longer than predicted by the disease-specific Graded Prognostic Index tool (estimated survival is 17 months with an interquartile range of 8-36 months) [7]. There is emerging evidence to support the role of ablative radiotherapy to RCC metastases, with excellent one-year survival rates, high rates of local control and very low rates of toxicity [6].

Repeat SRS for brain metastases has been previously demonstrated to be feasible, efficacious and tolerable [8]; however, the decision must be individualised with careful consideration to the location of disease and alternative treatment options available. This case report demonstrates that the same principle can be applied to patients with recurrent metastases to MC and highlights the feasibility of repeat SRS in this location. Hypofractionated SRS was used in this case as the morbidity of radionecrosis (RN) was outweighed by the implications of disease progression. Furthermore, systemic therapy had already been trialled and there was a 30-month interval between GK SRS treatments. RN rates are around 30% when using single-fraction re-treatments for intracranial targets [8]. Hypofractionation was utilised as it is thought to decrease the risk of RN, and thus may improve the therapeutic ratio in the re-irradiation setting [9]. Repeat GK SRS for acromegaly is also effective, with one study reporting tumour control in 83.3% with cranial nerve palsies in 14.3% and no reported temporal lobe necrosis [10]. Although repeat GK SRS was used effectively in this case for symptom relief, additional reports are required to assess the feasibility and side-effect profile of GK SRS for RCC metastatic to MC.

## Conclusions

RCC metastases to MC are a rare occurrence and typically present with facial pain and/or hypoesthesia. Previously reported treatments include surgical resection in combination with radiotherapy and systemic treatment. Our case has demonstrated both symptom relief and tumour control with SRS on two separate occasions in combination with systemic therapy, while avoiding the risks of surgical resection. Radiosurgery is a feasible treatment option for metastases to MC, particularly in the setting of recurrent local disease.
